# Caveolin-1-negative head and neck squamous cell carcinoma primary tumors display increased epithelial to mesenchymal transition and prometastatic properties

**DOI:** 10.18632/oncotarget.6099

**Published:** 2015-10-12

**Authors:** Alain C. Jung, Anne-Marie Ray, Ludivine Ramolu, Christine Macabre, Florian Simon, Fanny Noulet, Anne-Florence Blandin, Guillaume Renner, Maxime Lehmann, Laurence Choulier, Horst Kessler, Joseph Abecassis, Monique Dontenwill, Sophie Martin

**Affiliations:** ^1^ Université de Strasbourg, LBP, CNRS UMR 7213, Illkirch, France; ^2^ Laboratoire de Biologie Tumorale, EA 3430 Université de Strasbourg, CRLC Paul Strauss, Strasbourg, France; ^3^ Institute for Advanced Study and Center of Integrated Protein Studies, Technische Universität München, Department Chemie, Garching, Germany

**Keywords:** caveolin-1, integrins, head and neck cancer, metastasis

## Abstract

Distant metastases arise in 20-30% of patients with squamous cell carcinoma of the head and neck (HNSCC) in the 2 years following treatment. Therapeutic options are limited and the outcome of the patients is poor. The identification of predictive biomarkers of patient at risk for distant metastasis and therapies are urgently needed. We previously identified a clinical subgroup, called “R1” characterized by high propensity for rapid distant metastasis. Here, we showed that “R1” patients do not or at very low level express caveolin-1 (Cav1). Low or no expression of Cav1 is of bad prognosis. Disappearance of Cav1 enables cells to undergo epithelial-mesenchymal transition (EMT). EMT is associated with enhanced migration and invasion. Our study uncovered a new target, α_5_β_1_ integrin. Targeting α_5_β_1_ integrins might not only prevent metastasis of HNSCC but also delay the development of the primary tumor by reducing tumor cell viability. Cav1 detection might be taken into consideration in the future in the clinic not only to identify patients at high risk of metastasis but also to select patient who might benefit from an anti-integrin therapy.

## INTRODUCTION

Head and neck squamous cell carcinomas (HNSCC) arise from the epithelium of the upper digestive tract, including the oral cavity, the pharynx and the larynx. With over 600,000 new cases diagnosed each year, HNSCC is the 6^th^ most frequent malignancy in the world. Tobacco and alcohol are two important risk factors responsible for 72% of HNSCC cases [1]. Human papilloma virus (HPV) was recently shown as a causative agent for 26% of HNSCC [2]. In the absence of suitable biomarkers, therapeutic decisions are based on tumor localization and TNM staging (evaluation of tumor size (T), lymph node involvement (N) and the presence of distant metastases (M)). However, lesions with similar pathological features can differ in clinical outcome. Despite advances in the understanding of the molecular features of HNSCC along with improved treatments, the survival rate has been unchanged in the past 30 years. Lymph node metastasis (LNM) detected in 50% of patients at the time of diagnosis and distant metastases observed in about 20-30% of patients 2 years after the treatment of the primary tumor account for such persistent bad prognosis. The 5-year survival rate is 54% for patient with LNM and 32% for patients with distant metastasis [3]. Chemoradiation regimens have become a standard of care for the management of locally advanced HNSCC (for review [4] and references therein). However, the prognosis of recurrent/metastatic HNSCC remains poor, and few therapeutic options are available. Induction chemotherapy followed by chemoradiotherapy has been shown to have a benefit in the reduction of distant metastasis [5]. However, the therapeutic index (efficacy *vs*. toxicity ratio) of these protocols is poor. Thus, the identification of molecular prognostic biomarkers and potential alternative drug targets should help to guide therapeutic choices and identify patients who will most benefit from innovative therapeutic approaches and avoid aggressive treatments. To identify prognostic HNSCC molecular subgroups and potential biomarkers, we have recently conducted genome-wide integrated analysis of four omic data sets [6]. The integrated analysis of 3 omics (methylome, transcriptome and miRNome) uncovered a common robust subgroup of patients, called “R1”, which is characterized by high propensity for rapid distant metastasis. The way tumor cells interact with and modify their environment is a key issue in the acquisition of metastatic properties. In accordance, “R1” tumors are characterized by alterations of pathways involved in cell-cell adhesion, extracellular matrix (ECM), epithelial-to-mesenchymal transition (EMT), immune response and apoptosis [6]. Similarly, alterations of genes controlling adhesion, motility and invasiveness especially integrins were reported as the major molecular differences among metastatic and non-metastatic HNSCC tumors [7–9].

We previously reported that stronger adhesion, motility and invasiveness can be observed in glioblastoma expressing high levels of α_5_β_1_ integrins induced by the disappearance of Cav1 from cells [10, 11]. Subclassification of patients according to α_5_β_1_ integrins/Cav1 levels showed the existence of a cluster of patients exerting low Cav1/high α_5_β_1_ integrins levels [11] which was correlated with reduced overall survival [12]. Cav1 is the principal structural protein of caveolae able to control the subcellular distribution, activity and expression of molecules and receptors [13, 14]. Cav1 regulates therefore multiple cancer-associated processes including cellular transformation, tumor growth, cell migration and metastasis, cell death and survival, multidrug resistance and angiogenesis [15, 16]. Up-regulation of Cav1 is associated with metastatic disease progression of various cancers and appears therefore as a good promoter of tumor dissemination [17, 18, 19, 20]. In contrast, reduced expression of Cav1 was observed in HNSCC LNM [21, 22], correlates with breast cancer nodal metastasis [23] and is prometastatic in malignant melanoma [24]. Downregulation of Cav1 induced EMT and enhanced tumor cell invasion in various cancer cell lines [25].

We therefore addressed the role of Cav1 in the regulation of HNSCC metastasis in the metastatic-prone “R1” and the non metastatic prone “non-R1” subgroups and investigated the molecular mechanisms underlying the acquisition of the metastatic phenotype.

## RESULTS

### Cav1 expression is reduced in prometastatic tissue

**Figure 1:**
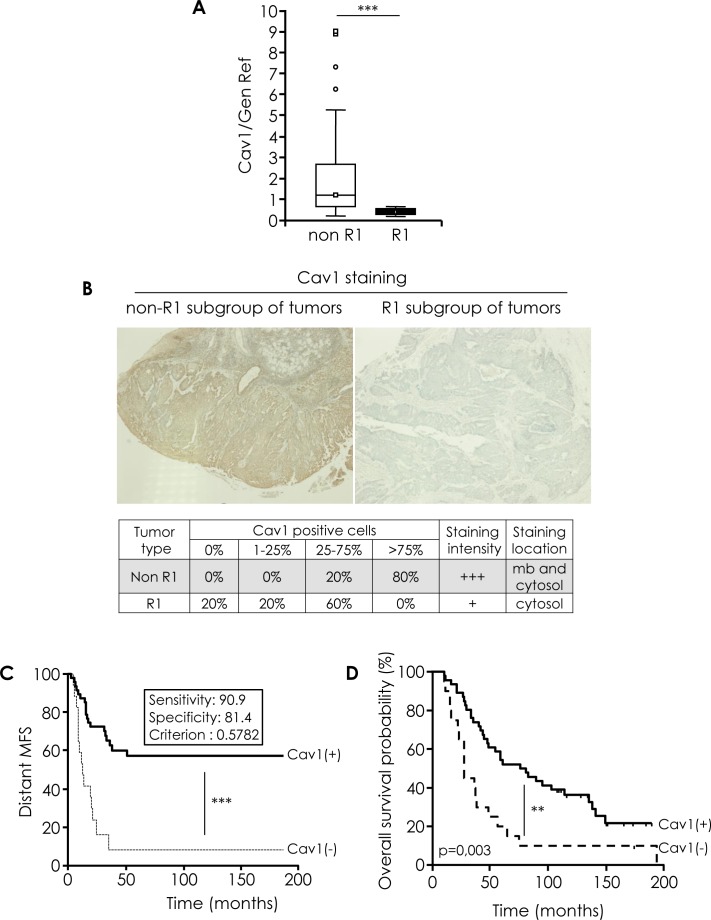
Cav1 expression in human HNSCC tissue specimens **A.** Quantified analysis of CAV1 transcripts determined in 11 primary tumor samples of patients that developed metastasis (“R1”) and 57 primary tumor samples of patients that did not developed metastasis (“non-R1”). The line within the bar represents the mean value and “o” represent individual data point. (***p < 0.001). **B.** Immunohistochemical analysis of Cav1 in R1 and non-R1 FFPE tissus (original magnification: X100). Table show % of non-R1 and R1 tumors with 0%, 1-25%, 25-75% and >75% Cav1-positive cells. **C.** Kaplan-Meier analysis of the distant metastasis-free survival (MFS) in patients stratified according Cav1 gene expression (CAV1(+) and CAV1 (−)). A cut-off value was determined for Cav1 gene expression (measured by qRT-PCR), corresponding to a 90.1% sensitivity and a 81.4% specificity with respect to the “R1” status (*i.e.* 90.1% of the “R1” lesions display a Cav1 expression level below this cut-off, and 81.4% of the “non-R1” tumours express Cav1 levels above this cut-off. More detail in suppl. material and methods). Samples were considered as Cav1-negative is the qRT-PCR value was ≤ to the cut-off. Shorter time to metastasis ***p < 0.001). **D.** Kaplan-Meier analysis of the overall survival (OS) in patients stratified according Cav1 gene expression (CAV1(+) and CAV1 (−)) as described in Fig 1C. Shorter time to death, **p = 0.003).

Analysis of Cav1 expression using a real-time qRT-PCR approach on RNA extracts from fresh-frozen tumours confirmed a strong and significant decrease of Cav1 expression in “R1” patients as compared to “non-R1” tumours (Figure 1A). Downregulation of Cav1 was further confirmed by immunohistochemical (IHC) analysis in the corresponding formalin fixed paraffin embedded (FFPE) tissues (Figure 1B). “Non-R1” tumors expressed Cav1 both at the plasma membrane and in the cytosol, with variable staining intensity. Cav1 staining was found to be largely weaker in “R1” tumour specimen. A semi-quantitative analysis of IHC staining was performed and tumours were classified in 4 categories according to the percentage of Cav1-positive carcinoma cells: 0%; 1-25%; 26-75%; > 75%. A majority of “non-R1” specimens showed > 75% of Cav1-positive cells, whereas no “R1” tumours belonged to that later category. This differences were found to be statistically significant (Figure 1B, χ^2^ test *p* = 0,007). Patients were stratified according to the Cav1 gene expression (see suppl. material and methods for cut-off value determination), and a Kaplan-Meier analysis of the distant metastasis-free survival (MFS) and of the overall survival (OS) were performed. Cav1 was found to have a prognosis value, since low caveolin expression correlated to adverse prognosis (shorter time to metastasis; *p* < 0.001; Figure 1C) and reduced OS (*p* < 0.005; Figure 1D).

### Low Cav1 expression increases cell motility and invasion

**Figure 2:**
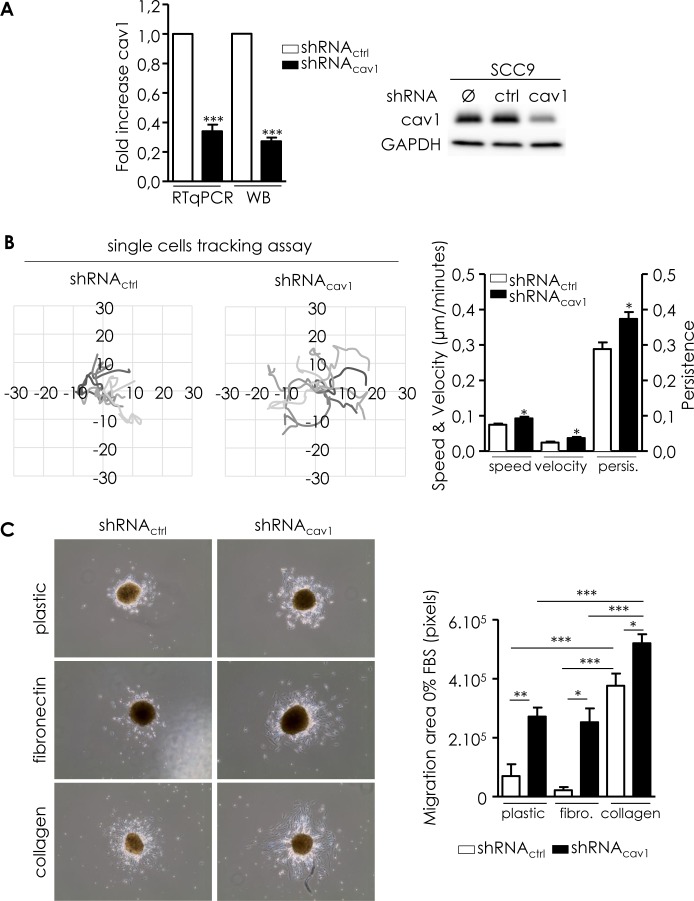

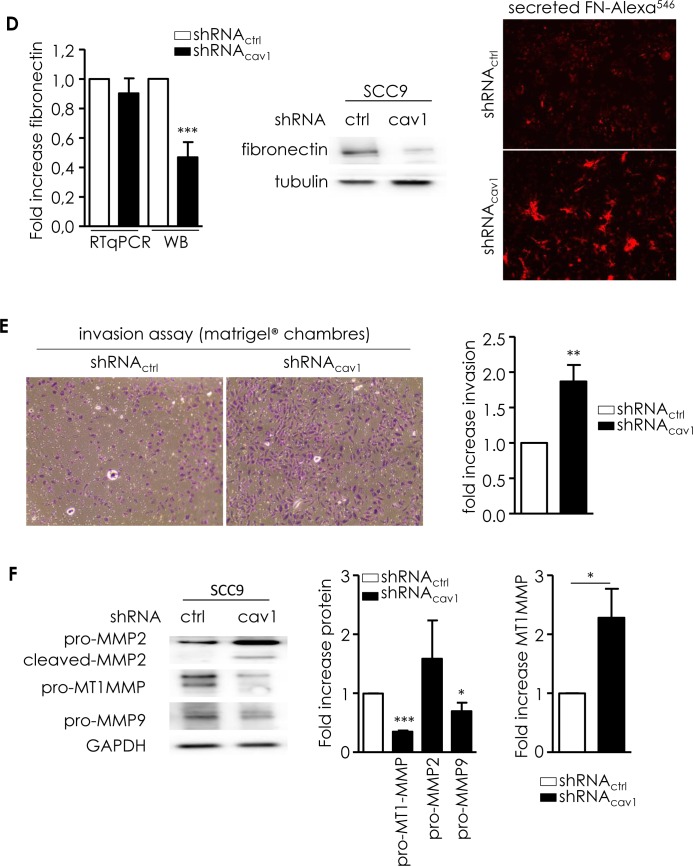
Reduction of Cav1 enables cells motile and invasive properties **A.** Quantitative determination of transcripts and protein expression of Cav1 in shRNA_ctrl_- or shRNA_cav1_-SCC9 cells using qRT-PCR with RNA18S as control and western blot using GAPDH as a loading control. Each bar represents the mean±SEM with ***p < 0.001. **B.** Analysis of single cell migration of shRNA_ctrl_- and shRNA_cav1_-transfected SCC9. Diagrams represent the migrating trajectories of cells covered during 6 hours by ten representative shRNA_ctrl_ and shRNA_cav1_-transfected SCC9. Each bar represents the mean±SEM of the speed, the velocity and the persistence recorded during 6 hours for each cell type (*n* = 4, *p < 0.05). **C.** Analysis of collective cell migration of shRNA_ctrl_- and shRNA_cav1_-transfected SCC9. Phase contrast images showing the evasion of shRNA_ctrl_- and shRNA_cav1_-transfected SCC9 from spheroids after 12 hours growth on plastic-, fibronectin- or collagen-coated cells. Each bar represents the mean±SEM area covered by cells evading from the spheroid (*n* = 7-9, *p < 0.05, **p < 0.01 and ***p < 0.001). **D.** Left panel: quantitative determination of transcripts and protein expression of fibronectin in shRNA_ctrl_- or shRNA_cav1_-SCC9 cells using qRT-PCR with RNA18S as control and western blot using tubulin as a loading control. Each bar represents the mean±SEM with ***p < 0.001, n = 8. Right panel: immunofluorescence analysis of extracellular secreted fibronectin by shRNA_ctrl_- or shRNA_cav1_-SCC9 cells. Images were taken at 4x magnification with an AXIO Zeiss microscope. **E.** Phase contrast images showing the invasion of shRNA_ctrl_- and shRNA_cav1_-transfected SCC9 through BioCoat Matrigel® invasion chambers. Cells were stained with crystal violet after 22 hours invasion. Each bar represents the mean±SEM fold increase in invasion (*n* = 6, **p < 0.01). **F.** MMPs protein levels were analyzed by western blot analysis using GAPDH as a loading control. Each bar represents the mean±SEM with *p < 0.05 and ***p < 0.001. MT1-MMP was analyzed in shRNA_ctrl_- or shRNA_cav1_-SCC9 cells using qRT-PCR with RNA18S as control. Each bar represents the mean±SEM with *p < 0.05, *n* = 4.

In order to evaluate the impact of the deregulation of Cav1 expression on the propensity of tumour cells to form distant metastasis, we generated a cell line expressing low level Cav1 and performed functional analysis (shRNA_cav1_-SCC9, Figure 2A). Migration was analyzed in single and collective cell migration assays. Individual cell migration was examined by live cell imaging in low density cell cultures (Figure 2B). Cell tracking measurements revealed that shRNA_cav1_-cells have a more persistent migration and a significant increase in the speed and velocity of migration than their control counterparts (Figure 2B). In other terms SCC9-shRNA_cav1_ explored larger areas than control cells. Consequences of Cav1 reduction were also determined in a collective 3D cell migration model using SCC9 spheroid. SCC9-shRNA_ctrl_ poorly migrated out of the spheres on plastic or fibronectin (FN)-coated dishes but strongly on collagen-coated dishes (Figure 2C). Independently of the matrix used, shRNA_cav1_-cells migrated out of aggregates more efficiently and covered an area significantly more important than control cells (Figure 2C). Data suggested that although collagen promoted strong evasion of cells, removal of Cav1 not only reinforced the process on collagen but also conferred cells the capacity to efficiently and significantly evade on a new matrix, FN. A strong secretion of FN into the extracellular environment was observed by microscopy in shRNA_cav1_-cells although no increased expression of FN was detected at the RNA and protein levels (Figure 2D).

To ascertain the role of Cav1 in the migration of HNSCC through 3D matrixes, matrigel^®^ invasion assays were performed. shRNA_cav1_-cells navigated through matrigel^®^ more efficiently than their control counterpart (Figure 2E). As invasion requires the degradation of underlying basement membrane, expression of matrix metalloproteases (MMPs) was analyzed. The reduction of Cav1 expression was associated with the apparition of an active cleaved MMP2 and a significant decrease of pro-MT1-MMP and pro-MMP9 (suggesting the activation of those MMP) in shRNA_cav1_-cells compared to control cells (Figure 2F). MT1-MMP transcript was significantly increased in shRNA_cav1_-cells (Figure 2F right panel). Altogether data showed that extinction of Cav1 in HNSCC resulted in enhanced migratory capacity and invasiveness.

### Low Cav1 expression induces the expression of specific integrins required for efficient migration and invasion

**Figure 3:**
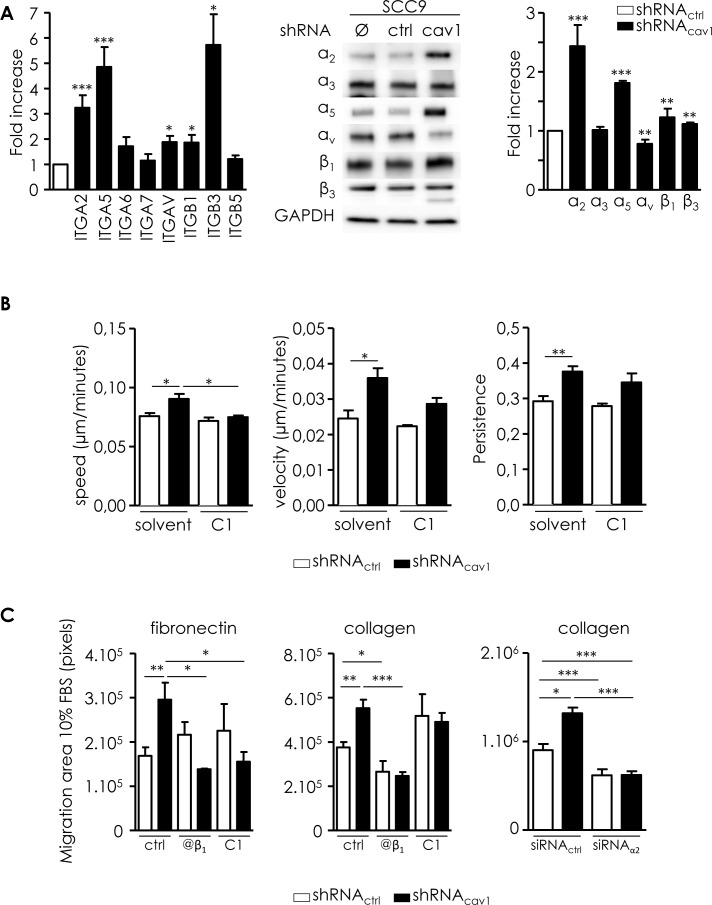

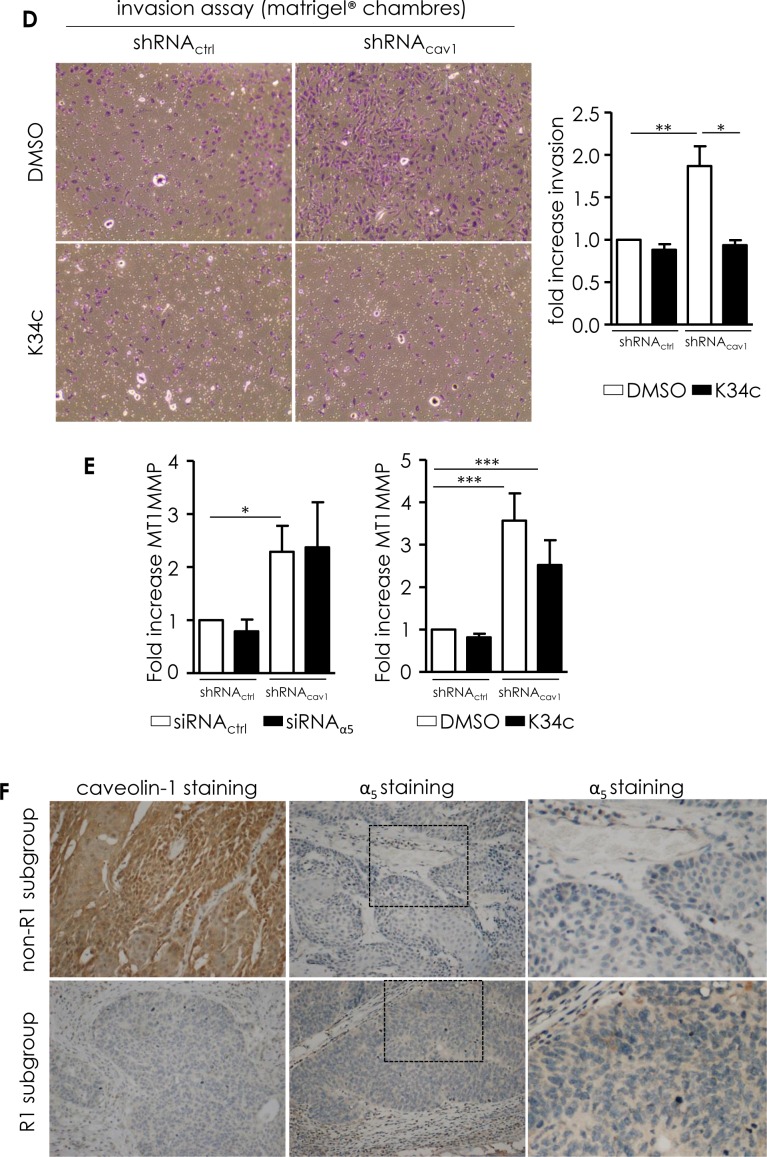
Integrins are involved in the motile and invasive properties of low Cav1 expressing cells **A.** Left panel: quantitative determination of transcripts expression of integrins (α_2_, α_5_, α_6_, α_7_, α_v_, β_1_, β_3_, β_5_ integrin subunits) in shRNA_ctrl_- or shRNA_cav1_-SCC9 cells using qRT-PCR with RNA18S as control. Each bar represents the mean±SEM with *p < 0.05, and ***p < 0.001. Right panel: integrin subunit protein levels (α_2_, α_3_, α_5_, α_v_, β_1_ and β_3_ integrin subunits) were analyzed by western blot analysis using GAPDH as a loading control. Each bar represents the mean±SEM with **p < 0.01 and ***p < 0.001. **B.** Analysis of single cell migration of shRNA_ctrl_- and shRNA_cav1_-transfected SCC9. Migrating trajectories of shRNA_ctrl_- and shRNA_cav1_-transfected SCC9 exposed to solvent or to a specific α_5_β_1_ integrin antagonist component 1 (C1, 20μmol/L) were recorded during 6 hours. Each bar represents the mean±SEM of the speed, the velocity and the persistence recorded during 6h for each cell type (*n* = 3-5, *p < 0.05 and **p < 0.01). **C.** Analysis of collective cell migration of shRNA_ctrl_ and shRNA_cav1_-transfected SCC9. Evasion of shRNA_ctrl_- and shRNA_cav1_-transfected SCC9 from spheroids exposed to solvent, to a specific α_5_β_1_ integrin antagonist component 1 (C1, 20 μmol/L) and to a β_1_ integrin specific blocking antibody OS2966 (10 μg/mL) were determined after 12 hours growth on fibronectin- or collagen-coated cells. Evasion of shRNA_ctrl_- and shRNA_cav1_-SCC9 transfected with siRNA_ctrl_ or siRNA_α2_ from spheroids were determined after 12 hours growth on collagen-coated cells. Each bar represents the mean±SEM area covered by cells evading from the spheroid (*n* = 4, *p < 0.05, **p < 0.01 and ***p < 0.001). **D.** Phase contrast images showing the invasion of shRNA_ctrl-_ and shRNA_cav1_-transfected SCC9 exposed to solvent or to a specific α_5_β_1_ integrin antagonist K34c (20 μmol/L) through BioCoat Matrigel^®^ invasion chambers. Cells were stained with crystal violet after 22 hours invasion. Each bar represents the mean±SEM fold increase in invasion (*n* = 4-6, *p < 0.05 and **p < 0.01). **E.** Quantitative determination of transcripts expression of MT1-MMP in shRNA_ctrl_- or shRNA_cav1_-SCC9 cells transfected with siRNA_ctrl_ and siRNA_α5_ integrin subunits or exposed to solvent and K34c (20 μmol/L) using qRT-PCR with RNA18S as control. Each bar represents the mean±SEM with *p < 0.05 and ***p < 0.001. **F.** Immunohistochemical (IHC) analysis of Cav1 (left panels) and α_5_ integrin subunit (middle panels) in representative “R1” and “non-R1” FFPE tissues are shown (original magnification: X200). Magnifications of insets shown in middle pictures are shown in the corresponding right panels (original magnification: X400).

Acquisition of invasiveness is crucial for metastatic dissemination. Invasion requires the modulation of cell-ECM adhesions mainly dependent on integrins. We previously reported that depletion of Cav1 significantly modified the expression of integrins in glioblastoma [10, 11]. shRNA_cav1_-cells expressed significantly more α_2_, α_5_,β_1_ and β_3_ integrin subunits than control cells at the RNA and protein level (Figure 3A). α_3_, α_5_, α_7_ and β_5_ integrin subunits were not significantly altered (Figure 3A). α_v_ integrin subunit was increased at the RNA level but decreased discreetly at the protein level in shRNA_cav1_ *versus* shRNA_ctrl_ (Figure 3A). Data suggested that two integrins were strongly increased following extinction of Cav1, α_2_β_1_ and α_5_β_1_ integrins. Those integrins were probably involved in the increase of cell migration on collagen and FN.

We ought to determine if resulting increased motile and invasive capabilities are dependent on those integrins. Cell tracking measurements revealed that compound 1, an antagonist of α_5_β_1_ integrin, totally abolished the increased speed of migration observed in shRNA_cav1_-cells without affecting basal migration of control cells (Figure 3B). Compound 1 did not affect velocity or persistence of cells as previously reported (Figure 3B, [26]). Data suggested that α_5_β_1_ integrin might be the integrin responsible for acquired motility of shRNA_cav1_-cells.

Consequences of α_5_β_1_ integrin inhibition (using an antibody blocking selectively the β_1_ integrin subunits and compound 1) were also determined in the collective cell migration model. Although migration of SCC9-shRNA_ctrl_ was not significantly altered by any drugs on FN-coated plates, evasion of SCC9-shRNA_cav1_ cells was abolished by the β_1_ antibody and by compound 1 (Figure 3C, left). Evasion of both cell lines was abolished by the β_1_ antibody but not compound 1 on collagen-coated plates (Figure 3C, middle). The involvement of α_2_β_1_ integrin in the increased motility of cells on collagen was determined after silencing α_2_β_1_ integrin (siRNAα_2_*versus* siRNA_ctrl_, suppl. data 1). In contrast with K34c that only affected the evasion of SCC9-shRNA_cav1_ on FN, silencing α_2_β_1_ integrin reduced the evasion of both SCC9-shRNA_ctrl_ (by 31.5%) and SCC9-shRNA_cav1_ (by 52.9%, Figure 3C, right). The increase of cell migration observed in SCC9-shRNA_cav1_ cells on both collagen and FN are linked to the increase of α_2_β_1_ and α_5_β_1_ integrin expression.

Blocking α_5_β_1_ integrin with another non peptidic α_5_β_1_ integrin antagonist, K34c, also totally abolished the invasive capacity of shRNA_cav1_-cells in 3D matrigel^®^ invasion assays (Figure 3D). K34c remained without effect on basal invasion of control cells. Silencing α_5_ integrin subunits or inhibiting α_5_β_1_ integrin using K34c did not affect either basal levels of MT1-MMP or MT1-MMP-induced expression in shRNA_cav1_-cells suggesting that the induction of MMPs was driven by Cav1 independently of integrins activation (Figure 3E). Data suggested that the extinction of Cav1 in HNSCC resulted in enhanced migratory capacity and invasiveness that is dependent of α_5_β_1_ integrin.

To ascertain that the alteration of α_5_β_1_ integrin expression observed following depletion of Cav1 had clinical significance, we performed IHC analysis in the corresponding FFPE tissues. “Non-R1” tumors which expressed Cav1 showed no or weak α_5_ integrin subunit expression. “R1” tumours specimen which express weak or no Cav1 exert α_5_ integrin subunit staining confirming *in vitro* data (Figure 3F).

### Low Cav1 expression forces HNSCC cells to undergo an EMT

**Figure 4:**
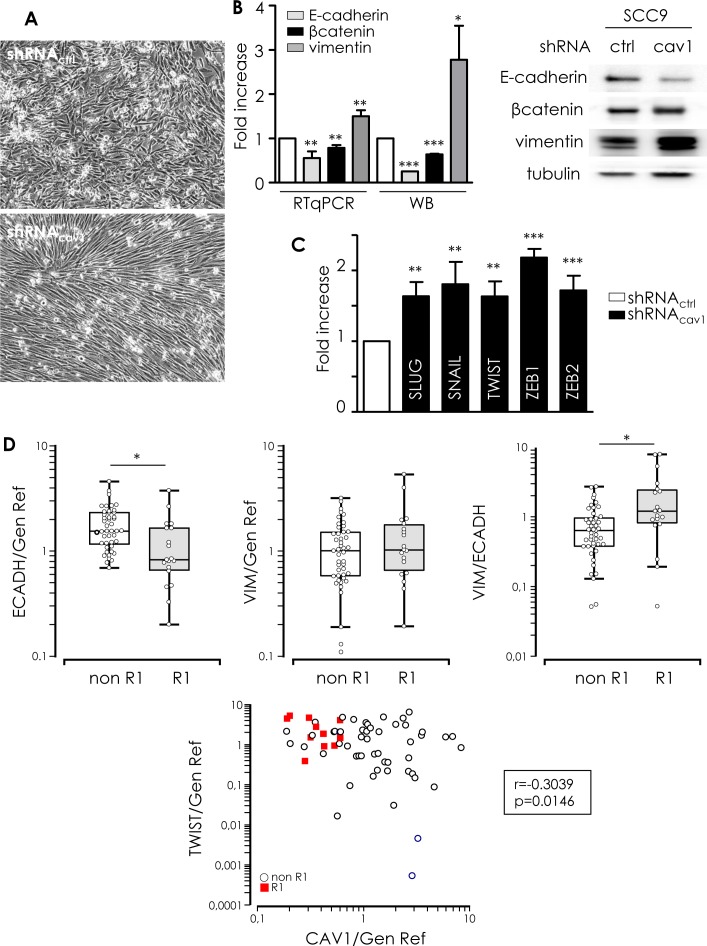

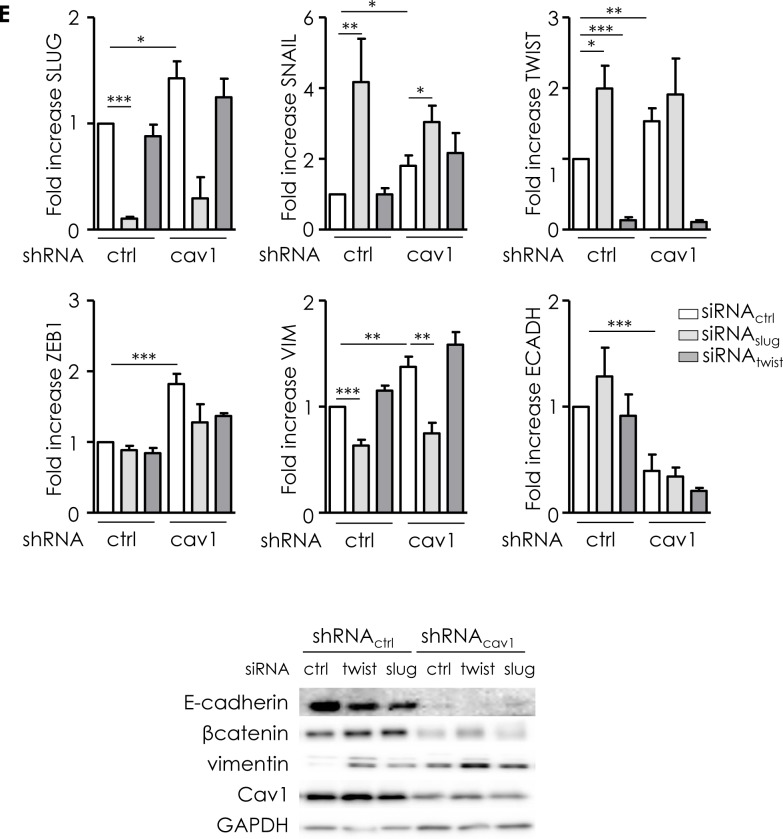

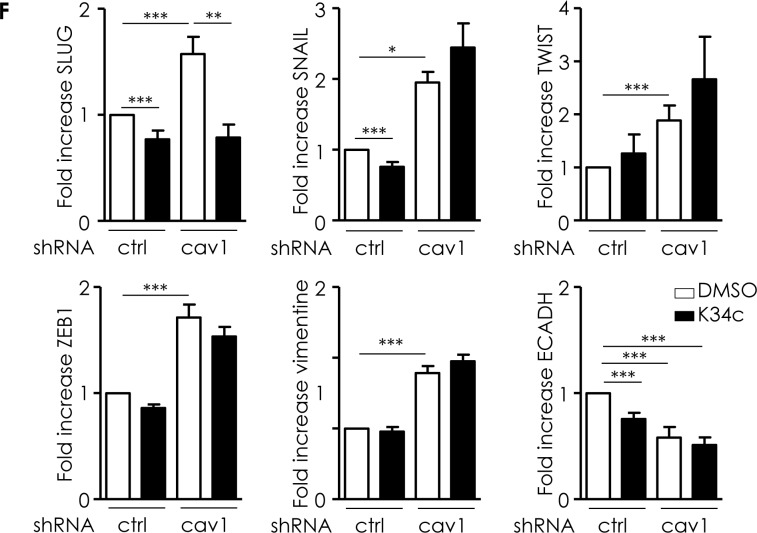
Low Cav1 expressing cells undergo an EMT **A.** Phase contrast images of stable SCC9 cells expressing shRNA_ctrl_ or shRNA_cav1_ in culture. **B.** Quantitative determination of transcripts expression of epithelial (E-cadherin and β-catenin) and mesenchymal (vimentin) markers in shRNA_ctrl_- or shRNA_cav1_-SCC9 cells using qRT-PCR with RNA18S as control and western blot using tubulin as a loading control. Each bar represents the mean±SEM with **p < 0.01 and *** < *p*0.001. **C.** Quantitative determination of transcripts expression of key regulator of E-cadherin and EMT (including Slug, Snail, Twist, ZEB1 and ZEB2) in shRNA_ctrl_- or shRNA_cav1_-SCC9 cells using qRT-PCR with RNA18S as control. Each bar represents the mean±SEM with **p < 0.01 and ***p < 0.001. **D.** Quantified analysis of E-cadherin, vimentin, Twist and Cav1 transcripts determined in primary tumor samples of patients that developed metastasis (“R1”) and primary tumor samples of patients that did not developed metastasis (“non-R1”). The line within the bar represents the mean value and “o” represent individual data point. (***p < 0.001). **E.** Quantitative determination of transcripts expression of Slug, Snail, Twist, Zeb1, vimentin and E-cadherin in shRNA_ctrl_- or shRNA_cav1_-SCC9 cells transfected by siRNA_ctrl_ or siRNA_SLUG_ or siRNA_TWIST_ using qRT-PCR with RNA18S as control. Each bar represents the mean±SEM with *p < 0.05, **p < 0.01 and *** < *p*0.001. **F.** Quantitative determination of transcripts expression of Slug, Snail, Twist, Zeb1, vimentin and E-cadherin in shRNA_ctrl_- or shRNA_cav1_-SCC9 cells exposed to DMSO or 20 μmol/L K34c using qRT-PCR with RNA18S as control. Each bar represents the mean±SEM with *p < 0.05, **p < 0.01 and *** < *p*0.001.

Enhanced migration and invasion, increased production of ECM and degradation of underlying basement membrane are multiple biochemical changes of a biological process allowing a polarized epithelial cell to acquire a mesenchymal phenotype so called EMT. Cav1 reduction in SCC9 resulted in a switch from a cuboid epithelial shape to a more fibroblastic appearance (Figure 4A). EMT was associated with a loss of E-cadherin and a reduction of β-catenin expression (Figure 4B). Simultaneously, the mesenchymal marker vimentin was significantly increased (Figure 4B). Analysis of the expression of key transcriptional regulators of EMT showed that shRNA_cav1_ cells expressed increased level of repressors of E-cadherin expression such as Slug, Snail, Twist, Zeb1 and Zeb2 (Figure 4C). We concluded that the extinction of Cav1 induces HNSCC cells to undergo EMT. We previously showed that the “R1” cluster of patient was characterized by alterations of pathways involved in EMT [6]. In accordance, a significant reduction of E-cadherin expression and an increase of the vimentin/E-cadherin ratio were found in “R1” patients compared to “non-R”1 tumours. In addition, “R1” patients exhibited significantly higher levels of Twist than “non-R1” tumours. Twist expression was found to be anti-correlated with Cav1 expression. Altogether, patient data confirmed *in vitro* studies showing an induction of EMT in “R1” tumors depleted in Cav1 (Figure 4D). To go further, we silenced both Slug and Twist to determine whether EMT induced by suppression of Cav1 is dependent on both transcription factors. Inhibition of Slug significantly increased SNAIL and TWIST expression in both shRNA_ctrl_ and shRNA_cav1_-SCC9 (Figure 4E). We hypothesise that such an increase in EMT markers after Slug inhibition might be due to the repression of Cav1 expression by Slug itself (suppl. data 2). Slug inhibition did not repress the increase of SNAIL, TWIST or ZEB1 in shRNA_cav1_-SCC9 and did not prevent the disappearance of E-cadherin in shRNA_cav1_-SCC9. However, Slug inhibition prevented the induction of vimentin in shRNA_cav1_-SCC9 (Figure 4E). Turning to Twist, its inhibition did not affect Cav1 expression or the increase of Slug, SNAIL, TWIST or ZEB1 in shRNA_cav1_-SCC9 and did not prevent the disappearance of E-cadherin in shRNA_cav1_-SCC9 (Figure 4E). Data suggest therefore that Slug contribute to the full transition as the absence of Slug prevents the appearance of the mesenchymal marker vimentin in shRNA_cav1_-SCC9.

α_5_β_1_ integrin was inhibited using K34c to determine its contribution to EMT in shRNA_cav1_ cells. K34c did not affect basal levels of TWIST, Zeb1 or vimentin but slightly inhibited Slug, Snail and E-cadherin. K34c only affected Slug induction in shRNA_cav1_-SCC9 cells (Figure 4F). Except for Slug, EMT gene expressions were activated independently of α_5_β_1_ integrin upon Cav1 removal.

### Low Cav1 expression is associated with a reduction of growth and survival capacities partially preserved by α_5_β_1_ integrins

**Figure 5:**
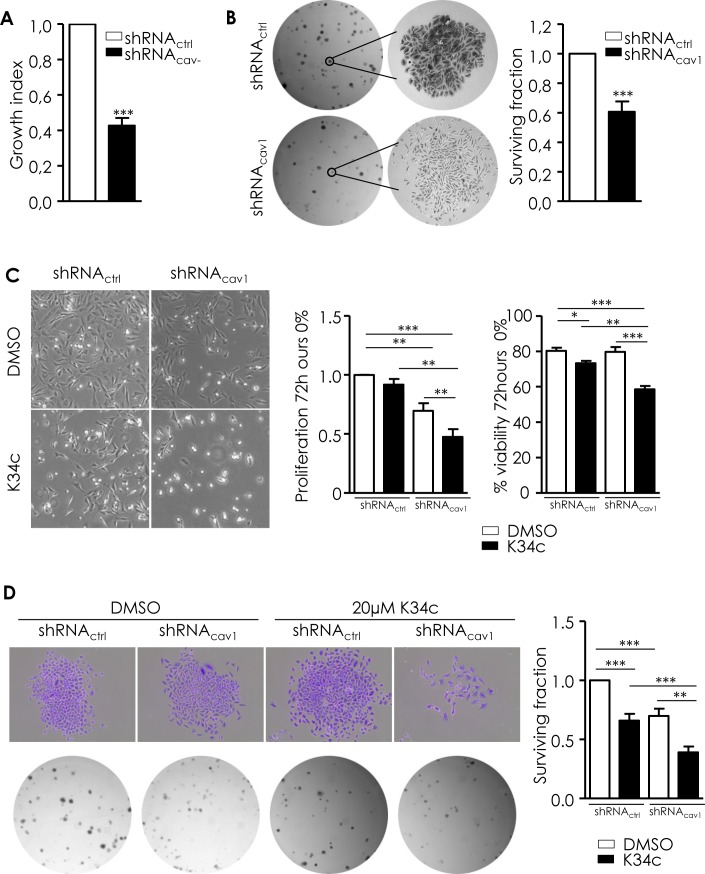
Low Cav1 expression is associated with a reduction of growth and survival capacities partially preserved by α_5_β_1_ integrins **A.** shRNA_ctrl_- and shRNA_cav1_-transfected SCC9 growth index was analyzed by regular cell counting using the TC20 counter (BioRad). Each bar represents the mean±SEM growth index of shRNA_cav1_- versus shRNA_ctrl_-transfected cells (*n* = 14, ***p < 0.001). **B.** ShRNA_ctrl_- and shRNA_cav1_- SCC9 were subjected to a clonogenic assay. Photograph represents phase contrast images of shRNA_ctrl_- and shRNA_cav1_- SCC9 colonies after staining with crystal violet. Each bar represents the mean±SEM surviving fraction of shRNA_cav1_- versus shRNA_ctrl_-transfected cells. Plating efficiency are 0.23±0.02, and 0.17±0.02 for shRNA_ctrl_ and shRNA_cav1_-transfected SCC9 respectively (*n* = 17, ***p < 0.001). **C.** Phase contrast images of stable shRNA_ctrl_ or shRNA_cav1_ SCC9 cells exposed to solvent or 20 μmol/L K34c up to 72 hours in 0% FBS containing medium. Each bar represents the mean±SEM number of cells and the percentage of proliferation and viability after trypan blue staining with *p < 0.05, **p < 0.01 and *** < *p*0.001. **D.** Phase contrast images of shRNA_ctrl_- and shRNA_cav1_- SCC9 colonies stained with crystal violet exposed to solvent or 20 μmol/L K34c up to 72 hours in 0% FBS containing medium. After refreshment, cells are allowed to grow 7 days in 10% FBS containing medium. Each bar represents the mean±SEM surviving fraction with **p < 0.01 and *** < *p*0.001.

Although reduction of Cav1 expression enabled cell invasive properties, one striking phenotype of shRNA_cav1_-SCC9 cells was a 57% reduction in the growth index of cells (Figure 5A) and a 40% reduction in the number of colonies in a clonogenic assay (Figure 5B). Although shRNA_cav1_-cells displayed reduced growth and fewer colonies, cells retained their ability to self-renew (right panel of photographs, Figure 5B) allowing maintenance of a population of tumorigenic cells. As α_5_β_1_ integrin played a crucial role in the acquisition of proliferative and clonogenic ability of Cav1-depleted glioma cells [10, 11], its role in the reduction of proliferation and clonogenicity observed in HNSCC cells with reduced expression of Cav1 was determined.

Involvement of α_5_β_1_ integrin in the growth and colony formation was analyzed using the selective antagonist K34c. K34c by itself did not affect basal proliferation but slightly reduced viability of control cells (Figure 5C). The inhibition of proliferation observed after Cav1 depletion was reduced even further in presence of K34c most probably because under such conditions viability of cells is significantly reduced (Figure 5C). As a consequence, surviving fraction of control cells as well as shRNA_cav1_ cells exposed to K34c was significantly reduced in clonogenic assays (Figure 5D). Depletion of α_2_β_1_ integrins did not affect clonogenic survival by itself and did not improve or worsened that of shRNA_cav1_-cells (suppl. data 3) suggesting that α_5_β_1_ integrins are the main integrins involved in the regulation of growth/survival/clonogenicity of HNSCC cells. Data suggested therefore that the reduction of growth and clonogenicity observed in shRNA_cav1_-cells was not due to α_5_β_1_ integrins. However as the inhibition of α_5_β_1_ integrin activity in those cells reduced even further the growth and the survival, α_5_β_1_ integrins seems crucial to maintain residual growth and survival of shRNA_cav1_ cells. To conclude, α_5_β_1_ integrins mediate both motile/invasive and survival properties of cells expressing low levels of Cav1.

## DISCUSSION

Therapeutic options for the management of metastatic HNSCC are limited, and the outcome of the patients is poor. Predictive biomarkers of metastatic tumors as well as therapies tied to genetic alterations involved in the metastatic process are urgently needed. Our study showed that the absence or low expression of Cav1 is observed in primary “R1” tumors with high propensity for distant metastasis. To date, only a few studies addressed the role of Cav1 in the metastatic process of HNSCC. However the reported data are inconsistent. Masuelli et al. showed that Cav1 is highly increased in well and poorly differentiated HNSCC and its overexpression was associated with LNM [18]. Increased expression of Cav1 was also observed in HNSCC cell lines selected for their aggressive metastatic phenotype [27]. Silencing Cav1 *via* lentiviral RNAi miR133a was shown to delay tumor growth *in vivo*, cell migration and invasion [28, 29]. On the contrary, significant decrease in Cav1 expression was observed in poorly differentiated HNSCC tumors and in LNM tissues of patients [21] without significant differences between primary tumors of patients who did or not have LNM. In an earlier study Zhang et al. reported a similar loss of Cav1 in cell lines generated from LNM tissues [9]. Here, we confirmed that low level of Cav1 correlates with adverse prognosis, reduced distant MFS and OS. We can therefore hypothesize that Cav1 expression is dramatically reduced in metastatic tissue as compared to primary tumour, as reported by Zhang et al. [22], but that this downregulation is also a hallmark of metastasis-prone HNSCC before extension to distant sites. To our knowledge, our study is the first showing rational and clinical evidence that no or low levels of Cav1 expression in primary HNSCC tumors might be predictive of patient with high metastasis risk as well as being of bad prognosis. However, due to a small cohort size and the retrospective nature of our study, our observations must be interpreted with caution. The prognostic power of Cav1 expression requires confirmation in a larger prospective clinical study in newly diagnosed HNSCC patients. IHC is a technique routinely used by pathologists for the diagnosis of solid tumors, we think that IHC staining of Cav1 might be easily translated in the future to the clinic. Patients with high nodal stage (N ≥ 2) tumours are at risk for distant metastasis. Monitoring the expression of Cav1 might be a useful tool for a more reliable evaluation of tumour propensity for metastasis, and to identify patients who could benefit from a new and more personalized medicine. For example, Mercier et al. reported that mTOR inhibitors such as rapamycin prevented tumor growth and increased survival rates in breast cancer patients with decrease stromal Cav1 expression [30]. In addition, our study showed that drugs targeting α_5_β_1_ integrins might also be effective in preventing HNSCC metastasis. Here we propose a biomarker that could rationally select specific subpopulation of HNSCC patients that might be responsive to more targeted therapy.

Consistently with patient data, decreased expression of Cav1 in HNSCC cell lines heightened pro-metastatic features reflected by increased migration, enhanced evasion and stronger invasion. Cav1 up-regulation enhanced cell mobility/migration, increased directional motility and invasion *in vitro* in Ewing's sarcoma tumors, hepatocellular tumors, lung carcinoma or even HNSCC [16]. In contrast, Cav1 promote proliferation whereas it suppressed migration and invasion of melanoma cells through the inhibition of the integrin/FAK/Src signaling pathway [24]. Silencing Cav1 in glioblastoma endowed cells mobility and invasion mediated by α_5_β_1_ integrins [11]. Downregulation of Cav1 induced EMT and enhanced tumor cell migration and invasion in various cancer cell lines [25]. Increased cell motility coupled to reduced growth observed here in HNSCC cells expressing reduced Cav1 expression suggest that Cav1 expression in primary HNSCC might contribute to tumor growth, whereas its loss is a key factor in metastatic progression. Acquisitions of migratory and invasive capacities are main characteristics of cells undergoing an EMT [31, 32]. Interestingly, beside alterations of pathways involved in cell-cell adhesion, ECM, immune response and apoptosis, “R1” tumors are characterized by alterations of the EMT pathway [6]. Cav1 deficiency induces EMT during peritoneal dialysis [33] and in HNSCC [21] as well as EndoMT in pulmonary endothelial cells [34]. In accordance, reduced expression of Cav1 in our model is linked to a loss of the cobblestone epithelial morphology, a switch from epithelial (E-cadherin) to mesenchymal (vimentin) marker expression and the expression of various transcription factors such as Twist, Snail, Slug, ZEB1 and ZEB2 known to orchestrate EMT. Induction of EMT following reduction of Cav1 expression might likely be due to the release of the TGFβ and/or the ERK1/2 pathways which are major inducers of EMT. Both pathways were shown to be activated in glioblastoma [10] and in human peritoneal cells following Cav1 silencing [33].

The way tumor cells interact with and modify their environment is crucial for the metastatic process. Cells undergoing type 3 EMT (carcinoma-metastatic transitions) are characterized by an integrin switch and a change in the extracellular matrix (for review [35]). Reduction of Cav1 expression in our model led to a change in the expression of integrins with the silencing of α_v_ integrins and the induction of α_5_ and α_2_ integrins. Although the regulation of α_5_β_1_ integrin expression deserves further studies, it is most probable that its overexpression in shRNA_cav-1_-cells is a consequence of EMT in those cells as inhibition of α_5_β_1_ integrin using K34c could not restore EMT markers at the level of control cells in shRNA_cav-1_-cells. EMT markers such as Zeb2 [36], Twist-AP1 [37] and SNAIL [38] were all described as direct inducers of α_5_ integrin expression. In addition α_5_β_1_ integrin expression was shown to be negatively regulated by E-cadherin [39]. If α_5_β_1_ integrins did not drive EMT, they seemed to play a crucial role in the motile and invasive phenotype acquired by cells expressing low level of Cav1 as their ligand FN clearly boosted the evasion of shRNA_cav-1_-cells out of the tumor spheres in contrast to control cells. Moreover α_5_β_1_ integrin inhibition totally prevented shRNA_cav-1_-cell migration, evasion and invasion. Although α_v_β_1_ and α_v_β_3_ integrins can also bind FN, it is unlikely that these integrins support the metastatic phenotype as the expression of the α_v_ integrin subunit is downregulated. α_5_β_1_ integrin was recently shown to have important role in tumor progression [40], metastasis [39] and/or resistance to therapies [41] in various cancers. Its involvement in the metastatic extension of HNSCC was suggested by Wang et al. who showed that FN clearly enhanced the invasive ability of β_1_ integrin expressing cells [42]. Overexpression of this integrin indicated a very poor prognosis in epithelial ovarian cancer [39] and glioblastoma [12]. In accordance, blocking α_5_β_1_ integrin activity using specific antagonist such as K34c and compound 1, used to successfully block this integrin in other model of solid tumors [10–12, 26, 43], strongly impaired single cell migration, evasion and invasion suggesting that α_5_β_1_ integrin is the main integrin involved in the motility and invasiveness of shRNA_cav-1_-cells. As shown in glioblastoma, depletion of Cav1 in HNSCC enhanced α_5_β_1_ integrin expression endowing cells with an aggressive phenotype [10, 11]. It is obvious that beside FN, collagen also efficiently promotes evasion out of the tumor mass. However, in primary tumors of metastatic cases of oral squamous cell carcinoma (OSCC), the expression of various ECM molecules such as laminin or collagen is decreased, while the expression of FN is increased when compared with the non-metastatic cases [44]. Anti-α_2_ blocking antibodies were reported not to affect the invasion of OSCC cells in contrast to those targeting α_5_ [45]. Coupled to the fact that α_5_β_1_ integrin antagonist totally blocked migration, evasion and invasion, data suggest that collagen/α_2_β_1_ integrin are not crucial for HNSCC metastasis in contrast to fibronectin/α_5_β_1_ integrin that seems to efficiently drive such process. However it remains true that if cells evolve in a collagen-rich environment targeting α_2_β_1_ integrins seems an efficient anti-metastasis strategy as evasion of the tumor mass is totally abolished by anti-β_1_ antibodies as well as by the silencing of the α_2_ integrin subunit. In addition, α_5_β_1_ integrin antagonist K34c significantly altered the viability of shRNA_cav-1_-cells and to a lesser extent of control cells that lead to a significant reduction of the clonogenic survival and therefore the self renewal capacity of SCC9 cells. Silencing α_2_β_1_ integrin has no effect. α_5_β_1_ integrin antagonist were previously reported to significantly alter the proliferation of glioblastoma cell lines [43, 46]. Data suggest therefore that α_5_β_1_ integrin plays a crucial role in the viability of HNSCC cells. In addition, α_5_β_1_ integrin was recently described as a master regulator of anoïkis in hepatocellular cancer [47]. In accordance, preliminary study showed that cells expressing low levels of Cav1 exerted strong resistance to anoïkis (not shown), a prerequisite for systemic circulation and secondary tumor formation in distant organs. Anti-α_5_β_1_ integrins therapy might therefore be a new therapeutic option for the “R1” subgroup of patient exerting low levels of Cav1 expression in order to prevent the development of metastasis but also the development of primary “R1” tumours.

In conclusion, our study showed for the first time that primary tumors from patients at high risk of distant metastasis express very low level of Cav1. It also clearly shows that low or no expression of Cav1 correlates with adverse prognosis. Disappearance of Cav1 enables cells to undergo EMT leading to increased motility, evasion and invasion capacities due to increased production of FN, an overexpression of its receptor α_5_β_1_ integrin and MMPs. Our study uncovered a new target that might open new therapeutic options for the clinical management of those patients. Targeting α_5_β_1_ integrins might not only prevent metastasis of HNSCC but also reduce the development of the primary tumor by reducing tumor cell viability. In conclusion, Cav1 detection might be taken into consideration in the future in the clinic not only to identify patients at high risk of metastasis but also to select patient who might benefit from an anti-integrin therapy.

## MATERIALS AND METHODS

### Cell lines, culture conditions

SCC9 cell lines were purchased from the ATCC and grown in DMEM-F12 (PAN Biotech) supplemented with 2mmol/L L-glutamine, 0.4μg/mL hydrocortisone (Sigma) and 10% FBS (Gibco). Cell lines were authenticated as described in suppl. material and methods.

### Human tissue samples

All tumor specimens (*N* = 68) were collected, stored and used with the patients' informed consent. Patients from the North-East region of France underwent initial surgical resection of their localized HNSCC between 1989 and 2002. Hematoxylin-eosin slides of paraffin-embedded tumor (FFPE) specimens were examined by two pathologists. All of the tumors were squamous cell carcinomas. For detailed patients demographics, see Suppl. Table 1. Gene expression assays were performed on total RNA from frozen tumor tissues as described in [6] (see also Suppl. Material and Methods). The following genes were evaluated: Cav-1, E-Cadherin, Twist, Vimentin (see suppl. Table 2 for the sequence of specific primers). The expression of Cav1 and α_5_ integrin subunit was evaluated by immunohistochemical analysis (IHC) as described in [48] (see also suppl. material and methods).

### Short interfering RNA and plasmids

ShRNA (OpenBiosystems) were transfected at 4 μg using 10μg/mL Arrest In™ and stable cell lines were selected with 1 μg/mL puromycine. SiRNA (Dharmacon) were transfected at 100 nmol/L using Lipofectamine™ 2000 (Invitrogen). Efficiency of protein silencing was determined by western blotting and RT-qPCR.

### Chemicals

The compound 34c (2-(S)-2,6-dimethlybenzamido)-3-[4-(3-pyridin-2-ylaminopro oxy)-phenyl] propionic acid) named K34c [12] was synthesized in our laboratory according the procedure described by Heckmann et al. [49]. Compound 1 [26] is one of the most selective non peptidic α_5_β_1_ integrin antagonist and was synthetized (as the compound 44) as described in Heckmann et al. [49]. OS2966, an anti-β_1_ integrin blocking antibody was kindly provided by OncoSynergy.

### Real-time quantitative PCR

RNA was extracted as previously described [11]. mRNA expression was evaluated by relative quantitative RT-qPCR analysis using the StepOne Plus (Applied Biosystems) FastSYBRGreen PCR detector with primers (Invitrogen) described in suppl. Table 2. Target cDNA expression was quantified using the comparative ΔΔCt method with 18S rRNA as an internal control.

### Western blot

Cells were lysed (1% Triton-X100, NaF 100 mmol/L, NaPPi 10 mmol/L, Na_3_VO_4_ 1mmol/L in PBS, supplemented with Complete anti-protease cocktail; Roche) and 1-20 μg of protein was separated by SDS-PAGE (BioRad) and transferred to PVDF membranes (Amersham). Blots were probed with various antibodies described in suppl. Table 3. Proteins were visualized with enhanced chemiluminescence using the LAS4000 imager and densitometry analysis was performed using the ImageQuant Software (GE Healthcare).

### Immunostaining of secreted fibronectin

Cells were plated at 2×10^5^ cells per chamber in Lab-Tek II CC2 chamber slide (Nunc) for 24 hours. Cells were fixed 10 minutes at room temperature with 4% PFA, blocked 1 hour with 1% BSA and stained for extracellular secreted fibronectin (1:200) 1 hour at room temperature. After extensive wash, anti-Alexa546 was incubated an additional 1 hour at room temperature. Slides were mounted using Prolon Gold antifade reagent (Invitrogen) and observed using a fluorescent microscope (AXIO, Zeiss) with a 4X magnification.

### Growth assay

shRNA_ctrl_ and shRNA_cav1_-transfected SCC9 were counted at each passage after plating at similar density using the TC20 cell counter (BioRad).

### Clonogenic assay

Clonogenic assays were performed as previously described [50]. Data were expressed as surviving fraction as described by Cosset et al. [10]. Plating efficiency are 0.23±0.02, and 0.17 ± 0.02 for shRNA_ctrl_ and shRNA_cav1_-transfected SCC9 respectively.

### Single cell tracking

Cells were seeded at 500 cells/well with 1μg/mL Hoechst3342 (Sigma) in μClear 96 well black plate (Dutscher) in L-15 medium (Sigma) supplemented with 10%FBS. After 24 hours, medium was removed and fresh medium containing DMSO or 20 μmol/L compound 1 was added. Migration was followed by fluorescent microscopy (IN Cell Analyzer 1000, GE Healthcare) during 6 hours. Analyses were performed only on cells tracked during the entire assay. Speed and trajectories were computed with Excel software as previously reported [26].

### Spheres evasion assay

Cells were seeded at 1×10^4^ per 100μL of regular culture supplemented with 20% methylcellulose per well in 96 well plate with round bottom for 12 hours to allow spheroid formation. Spheroids were collected and seeded in plastic, fibronectin- or collagen-coated (10μg/mL) 24 well plates (1 sphere/well) for additional 12 hours to allow evasion of cells from attached spheres. Pictures were taken using the Evos Core microscope (AMG) with a 10X magnification. Results were expressed as the area of evasion of cells from the area of the attached sphere (total area - sphere area) determined using the ImageJ software and results are expressed in pixels.

### Invasion assay

Lower chambers of BD BioCoat Matrigel invasion chamber (24 well, 8 μm pores, BD Biosciences) were filled with 750μl of 10% FCS culture medium. 2.5×10^4^ cells were seeded in the upper compartment in 0% FCS culture medium containing DMSO or 20 μM K34c for 22 hours according to manufacturer's instructions. Cells that had transmigrated and adhered to the lower surface were fixed in glutaraldehyde for 15 minutes, stained with 0.1% crystal violet for 30 minutes and counted. No cells were detected in the lower chamber.

### Statistical analysis

Data are represented as mean ± SEM. In all cases, n refers to the number of independent experiments. Statistical analyses were done with the Student's *t*-test or ANOVA where *p* < 0.05 was considered significant.

## SUPPLEMENTARY MATERIAL TABLES


