# Supplementary material for: Caveolin-1-negative head and neck squamous cell carcinoma primary tumors display increased epithelial to mesenchymal transition and prometastatic properties
Source: Oncotarget. 2015 Oct 12;6(39):41884–901. doi: 10.18632/oncotarget.6099 (PMC4747196; doi:10.18632/oncotarget.6099)

Caveolin-1-negative head and neck squamous cell carcinoma primary tumors display increased epithelial to mesenchymal transition and prometastatic properties

Supplementary Material

Cell Lines authentication

In order to check SCC9 cell line identity and stability, DNA was extracted and submitted to STR profiling. A set of 17 highly polymorphic microsatellites plus one Y-chromosomal microsatellite for gender determination were amplified by fluorescent PCR and analyzed by capillary electrophoresis using a Genetic Analyzer 3100 (Applied Biosystems). STR alleles were compared to the profiles (peak number / size) obtained upon initial culture after receipt of frozen SCC9 cells from the ATCC and confirmed cell line identity.

Human tissue samples

All tumor specimens (N=68) were collected, stored and used with the patients' informed consent. Patients from the North-East region of France underwent initial surgical resection of their localized head and neck squamous cell carcinoma (HNSCC) between 1989 and 2002 at the St Barbe Clinic (Strasbourg, France), followed by post-operative radiotherapy (61/68 cases) or chemo-radiotherapy (7/68 cases) at the Paul Strauss Cancer Center (Strasbourg, France) or the Civil Hospitals of Colmar or Mulhouse. Hematoxylin-eosin slides of paraffin-embedded tumor (FFPE) specimens were examined by two pathologists. All of the tumors were squamous cell carcinomas. The median age of the patients was 60 years (35-82 years). The inclusion criteria were: tumor localization (hypopharynx, oropharynx, oral cavity or tongue), any size (Tx), any lymph node status (Nx), no clinically-evident distant metastases (M0) by conventional clinical and diagnostic radiological examinations (computed tomography). The patients did not have any previous or

synchronous neoplasia. 11/68 tumour were previously classified as R1 tumours, and 57/68 tumours were classified as non-R1 (Jung et al, 2013).

Total RNA were extracted from frozen tumor tissues using DNA/RNA allprep minikits (Qiagen, France), according to the manufacturer's instructions. The integrity of extracted RNA was verified on an Agilent 2100 Bioanalyser (Agilent Technologies, Palo Alto, CA). RNA concentrations were measured using a ND-1000 NanoDrop spectrophotometer (Labtech, Palaiseau, France). 0.5 µg of extracted RNA was used for cDNA synthesis using the IScript™ cDNA Synthesis Kit (BioRad), according to the manufacturer's instructions. One µl of diluted cDNA corresponding to either 5 ng or 1.25 ng of reverse transcribed RNA, was analyzed with SyberGreen (Roche, Meylan, France), in duplicate, using the LightCycler 480 real-time PCR system. qRT-PCR data were analyzed using LightCycler® 480 software. Ct levels were normalized to the average Ct values of 2 internal controls (housekeeping genes): *UBB* (*Ubiquitine B*) and *RPLP0* (*Ribosomal Protein Large P0*). The following genes were evaluated: Cav-1, E-Cadherin, Twist, Vimentin (see suppl. Data for the sequence of specific primers.

The expression of markers was evaluated by immunohistochemical analysis (IHC) using a Ventana Autostainer Automat (Ventana Medical Systems). Slides were prepared from formalin-fixed paraffin-embedded tumor specimens. Five "R1" and 15 "non-R1" paraffin-embedded specimens corresponding to tumor samples that are adjacent to the samples used in the qRT-PCR analysis were analyzed. Slides were stained for Caveolin-1 (N-20:sc-894; Santa Cruz Biotechnology; dilution 1/50) and Integrin-α_5, (H-104:sc-10729; Santa Cruz Biotechnology; dilution 1/50) according to the manufacturers' instructions. Signals were revealed with the ultraView Universal DAB Detection Kit (Ventana Medical Systems), according to the manufacturer's instructions. All images were acquired on a Nikon Eclipse 80i microscope with 20X or 40X objectives. Contrast was uniformly adjusted on all images with Photoshop (Adobe) software.

For Kaplan-Meier analysis, a cut-off value was determined as follow: since Cav1 gene expression (measured by qRT-PCR) was found to be significantly different in “R1” and “non-R1” HNSCC, a ROC curve analysis of Cav1 gene expression was performed in order to evaluate its ability to discriminate between these two categories of tumours. A cut-off value corresponding to an optimal compromise between specificity and sensitivity was retained: this cut-off value corresponds to a 90.1% sensitivity and a 81.4% specificity with respect to the “R1” status (*i.e.* 90.1% of the “R1” lesions display a Cav1 expression level below this cut-off, and 81.4% of the “non-R1” tumours express Cav1 levels above this cut-off). Tumours that displayed Cav1 gene expression above and below the cut-off value were stratified in the Cav1 (+) and Cav1 (-) categories, respectively.

Supplementary table 1 :
Detailed demographics and clinical features of the cohort of 68 HNSCC.

	**HNSCC patients N=68**
**Gender :**	
Male	61 (90%)
Female	7 (10%
**Age** (median=60 years):	
<60 years old	35 (51%)
≥60 years old	34 (49%)
**Tobacco smoking:**	
No	2 (3%)
Yes	52 (76%)
NA	14 (21%)
**Alcohol drinking:**	
No	1 (1%)
Yes	52 (76%)
NA	15 (2%)
**Tumour localization**	
Gum, mouth floor, oral cavity	4 (6%)
Hypopharynx	22 (32.5%)
Oropharynx	37 (54.5%)
Tongue	3 (4%)
Poorly defined localization	2 (3%)
**Pathological lymph node staging (pN):**	
N0	13 (19%)
N1	8 (12%)
N2	40 (59%)
N3	7 (10%)
**Pathological tumour size staging (pT):**	
T1	/
T2	30 (44%)
T3	29 (43%)
T4	9 (13%)
**Tumour stage**	
Stage I	/
Stage II	5 (7%)
Stage III	14 (21%)
Stage IV	49 (72%)
**Tumour histology:**	
Well differentiated	13 (19%)
Moderately differentiated	27 (40%)
Poorly differentiated	27 (40%)
Non differentiated	1 (1%)
**Treatment**	
Surgery + radiotherapy	61 (90%)
Surgery + chemoradiotherapy	7 (10%)
**R1 status**	
R1	11 (16%)
Non-R1	57 (82%)

Supplementary table 2. Probes used for real-time RT-qPCR analysis.

Probes	Forward	Reverse
18s rRNA	5'-TGTGGTGTTGAGGAAAGCAG-3'	5'-TCCAGACCATTGGCTAGGAC-3'
CAV1	5'-ACCGCGACCCTAAACACCTC-3'	5'-CCTTCCAAATGCCGTCAAAA-3'
ITGA2	5'-GCAGGACAGAAATCACAGTTCA-3'	5'-GCAACAAAGTGAGTGCTTTCTC-3'
ITGA5	5'-GGGCAGCAGGACAGGGTTAC-3'	5'-GCCTTGCCAGAAATAGCTTCC-3'
ITGA6	5'-ATTCTCATGCGAGCCTTCAT-3'	5'- GAAACACAGTCACTCGAACC-3'
ITGA7	5'-CGACACGGAATTCCAACCT-3'	5'-CATCAGCTCCAGGCCAAT-3'
ITGAV	5'-AGGTGCCTACGAAGCTGAGC-3'	5'-AAGGCTTCATTGTTTCGGACA-3'
ITGB1	5'-CAAA TTGTGGGTGGTGCACA-3'	5'-TGGAGGGCAACCCTTCTTT-3'
ITGB3	5'-CTCAGCGACAAGGGCTCTG-3'	5'-TCTTCGAATCATCTGGCCG-3'
ITGB5	5'-CGAGTTCGGCAAGATCTATGG-3'	5'-CATGGCCTGAGCAGAGGACT-3'
MT1MMP	5'-GGAACCCTGTAGCTTTGTGTCTGTC-3'	5'-TGAGGGTCCTGCCTTCAAGTG-3'
Ecadherin	5'-TACACTGCCCAGGAGCCAGA-3'	5'-TGGCACCAGTGTCCGGATTA-3'
Bcatenin	5'-GCTGAAGGTGCTATCTGTCTGCTC-3'	5'-TGAACAAGACGTTGACTTGGATCTG-3'
Vimentin	5'-TGAGTACCGGAGACAGGTGCAG-3'	5'-TAGCAGCTTCAACGGCAAAGTTC-3'
Fibronectin	5'-TGCCTTGCACGATGATATGGA-3'	5'-CTTGTGGGTGTGACCTGAGTGAA-3'
Snail	5'-GACCACTATGCCGCGCTCTT-3'	5'-TCGCTGTAGTTAGGCTTCCGATT-3'
Slug	5'-ATGCATATTCGGACCCACACATTAC-3'	5'-AGATTTGACCTGTCTGCAAATGCTC-3'
Twist	5'-GGAGTCCGCAGTCTTACGAG-3'	5'-TCTGGAGGACCTGGTAGAGG-3'
Zeb1	5'-GAAAGTGATCCAGCCAAATGGAA-3'	5'-TTTGGGCGGTGTAGAATCAGAG-3'
Zeb2	5'-AAATGCACAGAGTGTGGCAAGG-3'	5'-CTGCTGATGTGCGAACTGTAGGA-3'

Supplementary table 3. Antibodies used for Western blot analysis and immunostaining.

Antibodies	Dilution / Origin
α_1	1:200, R-164, Santa Cruz Biotechnologies
α_2	1:200, N-19, Santa Cruz Biotechnologies
α_3	1:1000, AB1920, Chemicon
α_5	1:1000, AB1928, Chemicon
α_6	1:1000, 3750, Cell Signaling
α_V	1:3000, AB1930, Chemicon
β_1	1:1000, AB1952, Chemicon
β_3	1:1000, 4702, Cell Signaling
E-cadherin	1:1000, BD Bioscience
βcatenin	1:1000, BD Bioscience
vimentin	1:1000, H-84 , Santa Cruz Biotechnologies
fibronectin	1:1000, BD Transduction Laboratories
caveolin-1	1:1000, N-20, Santa Cruz Biotechnologies
MMP2	1:1000, 4022, Cell Signaling
MMP9	1:1000, 3852, Cell Signaling
MT1-MMP	1:1000, 13130, Cell Signaling
tubulin	1/1000, Mob286, DBS
GAPDH	1:10000, MAB374, Chemicon
Goat anti-mouse HRP-conjugated	1:10,000, Promega
Goat anti-rabbitHRP-conjugated	1:10,000, Promega

Supplementary data 1

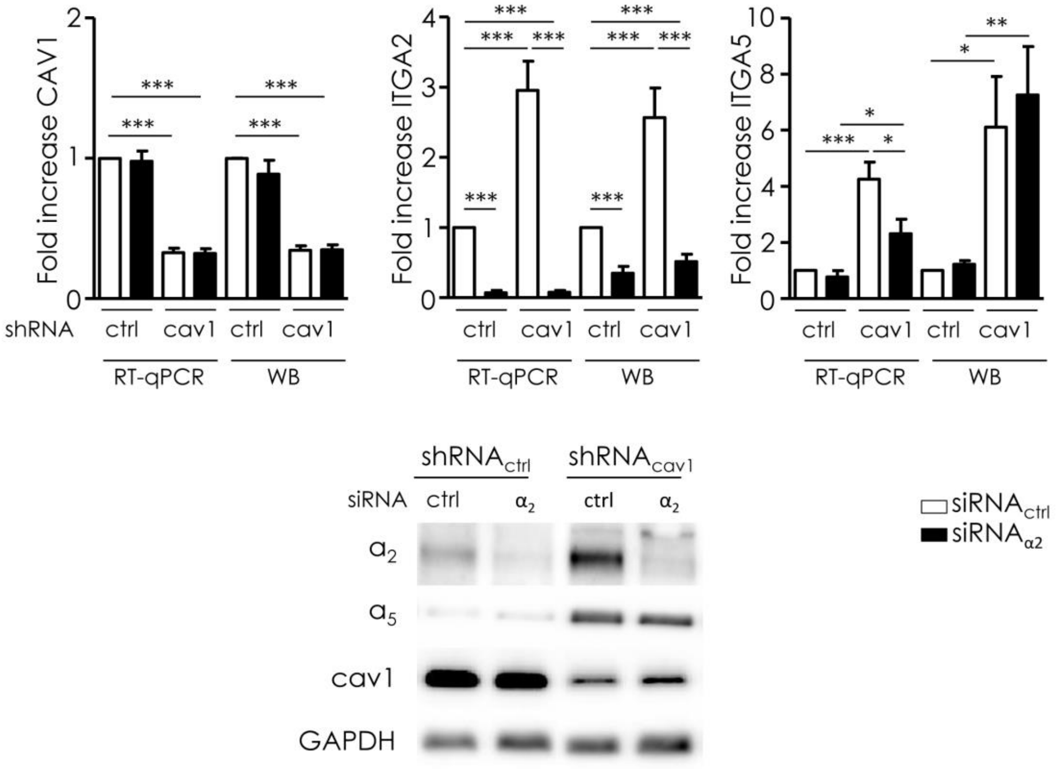

Supplementary data 2

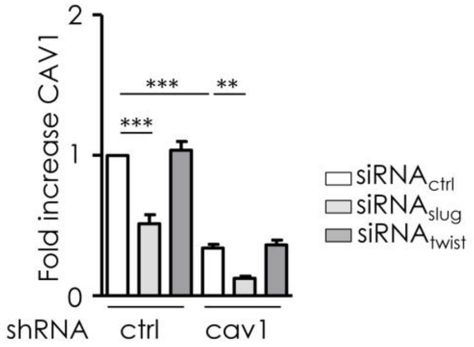

Supplementary data 3.

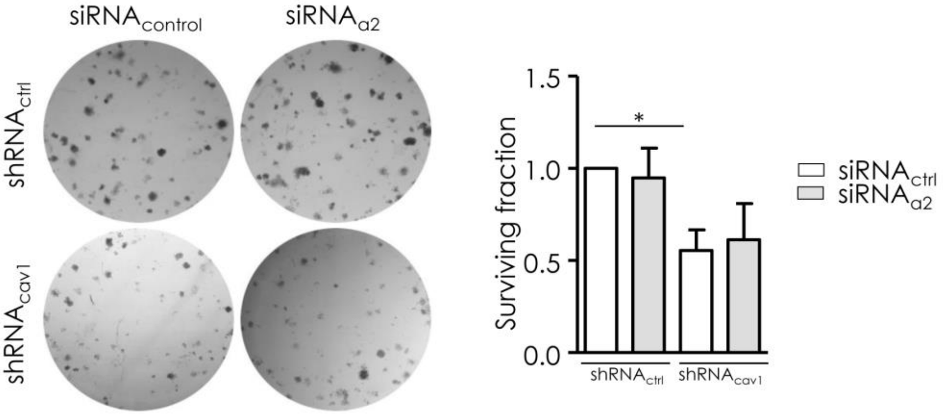

Supplement: Supplementary file 1 [file oncotarget-06-41884-s001.pdf]
